# Characterization of self-generated variants in *Pseudoalteromonas lipolytica* biofilm with increased antifouling activities

**DOI:** 10.1007/s00253-015-6865-x

**Published:** 2015-08-12

**Authors:** Zhenshun Zeng, Xing-Pan Guo, Baiyuan Li, Pengxia Wang, Xingsheng Cai, Xinpeng Tian, Si Zhang, Jin-Long Yang, Xiaoxue Wang

**Affiliations:** Key Laboratory of Tropical Marine Bio-resources and Ecology, Guangdong Key Laboratory of Marine Materia Medica, RNAM Center for Marine Microbiology, South China Sea Institute of Oceanology, Chinese Academy of Sciences, Guangzhou, 510301 People’s Republic of China; University of Chinese Academy of Sciences, Beijing, 100049 China; Shanghai Ocean University, Shanghai, China

**Keywords:** *Pseudoalteromonas*, Biofilm variant, Exopolysaccharide, Antifouling

## Abstract

*Pseudoalteromonas* is widespread in various marine environments, and most strains can affect invertebrate larval settlement and metamorphosis by forming biofilms. However, the impact and the molecular basis of population diversification occurring in *Pseudoalteromonas* biofilms are poorly understood. Here, we show that morphological diversification is prevalent in *Pseudoalteromonas* species during biofilm formation. Two types of genetic variants, wrinkled (frequency of 12 ± 5 %) and translucent (frequency of 5 ± 3 %), were found in *Pseudoalteromonas lipolytica* biofilms. The inducing activities of biofilms formed by the two variants on larval settlement and metamorphosis of the mussel *Mytilus coruscus* were significantly decreased, suggesting strong antifouling activities. Using whole-genome re-sequencing combined with genetic manipulation, two genes were identified to be responsible for the morphology alternations. A nonsense mutation in *AT00*_*08765* led to a wrinkled morphology due to the overproduction of cellulose, whereas a point mutation in *AT00*_*17125* led to a translucent morphology via a reduction in capsular polysaccharide production. Taken together, the results suggest that the microbial behavior on larval settlement and metamorphosis in marine environment could be affected by the self-generated variants generated during the formation of marine biofilms, thereby rendering potential application in biocontrol of marine biofouling.

**Electronic supplementary material:**

The online version of this article (doi:10.1007/s00253-015-6865-x) contains supplementary material, which is available to authorized users.

## Introduction

Biofilm formation is an integral component of the bacterial life cycle and a key factor for bacterial survival in diverse environments ( Hall-Stoodley et al. 2004). Marine biofilms (microbial mats, floating biofilms, and aggregates) are unique ecological niches within which multiple organisms, such as invertebrates, sessile plants, and animals, attach and grow on a surface submerged in the ocean via a dynamic process (Cooksey and Wigglesworthcooksey 1995; Lee et al. 2014). Marine biofilms play important roles in various biological and ecological processes, including carbon cycling (Jiao et al. 2014; Mounier et al. 2014), nitrogen fixation (Barlett and Leff 2010), sulfate reduction (Santegoeds et al. 1998), and larval recruitment of marine invertebrates (Huang et al. 2007; Qian et al. 2007; Wang et al. 2012; Yang et al. 2013).

Marine *Pseudoalteromonas*, an important bacterial genus that is found in oceans throughout the world, has gained attention due to its ecological significance and its capacity to synthesize bioactive compounds by forming biofilms (Bernbom et al. 2011; Holmstrom and Kjelleberg 1999). Over 90 *Pseudoalteromonas* genomes have been sequenced with the aim of exploring the adaptive strategies used by these bacteria in various habitats (Medigue et al. 2005; Qin et al. 2011; Thomas et al. 2008; Yu et al. 2013; Zeng et al. 2014). The attached lifestyle and exopolysaccharide (EPS) richness of these strains suggest that they play important roles in marine ecosystems due to their ability to form marine biofilms (Poli et al. 2010). In marine environments, the undesirable accumulation of organic molecules and microorganism on submerged surfaces is termed biofouling (Patel et al. 2003). Marine invertebrates including mussels are typical members of fouling communities throughout the world, mainly due to high tolerance to various environment conditions and enormous reproductive potential (Yang et al. 2008). For *Pseudoalteromonas*, several species have been shown to induce or inhibit invertebrate larval settlement and metamorphosis of marine invertebrates (Dobretsov et al. 2006; Hadfield 2011; Holmstrom et al. 1996, 2002; Shikuma et al. 2014; Yang et al. 2013). However, little attention has been given to explore the molecular basis of bacterial mediation of larval settlement and metamorphosis using isogenic *Pseudoalteromonas* species.

Studies of commensal and pathogenic bacteria have shown that within-population diversification, especially diversification that occurs during biofilm formation, can help explain the adaptive strategies used by bacteria to cope with heterogeneous environments (Devries and Ohman 1994; Moyano et al. 2007; Rice et al. 2009; Webb et al. 2004). During biofilm formation, cells with diverse genotypes and phenotypes are generated (McElroy et al. 2014; Stewart and Franklin 2008). Much of this diversification occurring in biofilms (including laboratory-growing biofilms) can be explained by recognizing the microscale heterogeneity in environmental factors that is present within a biofilm (Rainey and Travisano 1998). However, studies of genetic basis of within-population diversification of *Pseudoalteromonas* and the ecological consequences of diversification have been very limited, mainly due to the difficulty of genetic manipulation of bacteria within this genus.

In this study, we found that the diversification of colony morphology regularly occurs in most *Pseudoalteromonas* species during biofilm formation. Nine out of twelve species display self-generated variation, including wrinkled or translucent variants, suggesting that diversification in colony morphology is common in this genus. Of the species studied, *Pseudoalteromonas lipolytica* produced the most distinct and diverse variants. We recently sequenced and assembled the complete genome of *P. lipolytica* SCSIO 04301 (Zeng et al. 2014) and have also successfully developed a gene deletion and complementation system in this strain (Wang et al. 2015). Therefore, we selected *P. lipolytica* as a model to investigate the potential ecological significance of the variants produced during biofilm formation and to explore the genetic changes that lead to the alterations of colony morphology by whole-genome re-sequencing.

## Materials and methods

### Strains, plasmids, and growth conditions

The bacterial strains and plasmids used in this study are listed in Table 1, and the sequences of the primers used are listed in Table S1. *P. lipolytica* SCSIO 04301 was isolated from sediment taken from a depth of 63 m in the South China Sea. In this part of the South China Sea, the temperature is generally 24–29 °C at a depth of 50–100 m and the salinity is approximately 33.2–34.2 ‰ (Huang 1988). *P. lipolytica* SCSIO 04301 has been deposited in the Guangdong Microbiology Culture Center under the accession number GIMCC 1.828. The whole-genome shotgun project has been deposited at DDBJ/EMBL/GenBank under the accession numbers JDVB00000000. *Escherichia coli* strains were grown in LB at 37 °C, and 0.3 mM DAP (2,6-diamino-pimelic acid) was added to the culture medium to culture *E.coli* WM3064. *Pseudoalteromonas* strains were grown in 2216E or Seawater Luria-bertani (SW-LB medium) (1 % tryptone and 0.5 % yeast extract dissolved in seawater) at 25 °C. Chloramphenicol (30 μg/mL) was used to maintain pBBR1MCS-based plasmids, and kanamycin (50 μg/mL) and erythromycin (25 μg/mL) were used to maintain pK18mobsacB-ery-based plasmids in *E. coli* hosts and in *Pseudoalteromonas* hosts, respectively.

**Table 1:** Bacterial strains and plasmids used in this study

Strains or plasmids	Relevant characteristics	Source
*E.coli* strains		
WM3064	RP4(tra) in chromosome, DAP^−^	(Dehio and Meyer 1997 **)**
Isogenic mutants of *Pseudoalteromonas lipolytica* SCSIO 04301		
Δ*08765*	In-frame deletion of *AT00*_*08765*	This study
Δ*17125*	In-frame deletion of *AT00*_*17125*	This study
Δ*17170*	In-frame deletion of *AT00*_*17170*	This study
Δ*17220*	In-frame deletion of *AT00*_*17220*	This study
Δ*08765* Δ*bcsZB*	In-frame deletion of *AT00*_*08765*, *bcsZ*, *bcsB*	This study
Plasmid		
pK18mobsacB-ery	pK18mobsacB containing the erythromycin-resistant gene from pHT304, Kan^r^, Ery^r^	(Wang et al. 2015)
pK18mobsacB-ery-*08765*	Recombinant plasmid for deleting *AT00*_*08765*	This study
pK18mobsacB-ery-*17125*	Recombinant plasmid for deleting *AT00*_*17125*	This study
pK18mobsacB-ery-*17170*	Recombinant plasmid for deleting *AT00*_*17170*	This study
pK18mobsacB-ery-*17220*	Recombinant plasmid for deleting *AT00*_*17220*	This study
pK18mobsacB-ery-*bcsZB*	Recombinant plasmid for deleting *bcsZB*	This study
pBBR1MCS-Cm	Broad-host-range vector containing the chloramphenicol-resistant gene from pWD2	This study
pBBR1MCS-*08765*	*AT00*_*08765* cloned into pBBR1MCS-Cm	This study
pBBR1MCS-*17125*	*AT00*_*17125* cloned into pBBR1MCS-Cm	This study

Erythromycin (25 μg/mL) and chloramphenicol (30 μg/mL) were used to maintain the pK18mobsacB-ery and pBBR1MCS-Cm plasmids, respectively

### Isolation of biofilm variants

Biofilm was incubated without shaking to produce a spatially heterogeneous environment as previously reported (Armitano et al. 2014; Rainey and Travisano 1998). Liquid-air biofilms were grown in 2216E or SW-LB medium in glass beakers and test tubes without shaking for an indicated time at 25 °C. Pellicles were assayed by visual inspection of the air-liquid interface of the standing culture. Morphology was observed and photographed every day during the culturing. For the isolation and scoring of variants, biofilms were harvested and uniformly homogenized and then diluted in 10-fold serial dilution steps into seawater. At the same time, planktonic cultures were maintained as biofilms for comparison. The dilutions were plated on SW-LB agar plates so as to obtain 30–300 colonies on each plate. A total of 1000 colonies were examined and measured to calculate the variation. At least three independent experiments were conducted and evaluated.

### Spawning and larval culture of mussels

Adults of *Mytilus coruscus* were collected from the coast of Shengsi, Zhoushan (122° 44′ E; 30° 73′ N), China. After spawning, *M. coruscus* larvae were cultured as previously described (Wang et al. 2012; Yang et al. 2008). Briefly, mussels were transferred to individual 2-l glass beakers when they were ready to spawn. Sperms and eggs were collected using a glass pipette and were transferred to a beaker containing filtered seawater (FSW; acetate-fiber filter 1.2-μm pore size). Fertilization was achieved by gently mixing eggs with a sperm suspension in FSW and maintained undisturbed for 20 min. Fertilized eggs were filtered onto a nylon plankton net (mesh size 20 μm) to remove excess sperm, washed thoroughly with FSW, and left undisturbed for 2 days in an incubator maintained at 18 °C. After 2 days, swimming straight-hinge veliger larvae were collected, washed gently with FSW, and cultured in 2-l glass beakers at an initial density of 5 larvae mL^−1^. Larvae were fed a diet of *Chaetoceros gracilis* at 5 × 10^4^ cells/mL/day. The culture water was changed every 2 days and the temperature was maintained at 18 °C. Larvae were cultured to the pediveliger stage of growth and were ready for use in settlement and metamorphosis bioassays.

### Larval settlement and metamorphosis bioassay

Biofilms of *P. lipolytica* and the two variants were prepared following a previously described method (Yang et al. 2013). Briefly, each strain was cultured in 2216E at 25 °C for 48 h and then cells were harvested by centrifugation at 1600*g* for 15 min. Cell pellet was washed three times by autoclaved filtered seawater (AFSW), and final cell density was adjusted to 10^6^~10^7^ colony forming unit (CFU)/mL. Cell suspension was transferred to sterile glass Petri dishes, each of which contained one piece of sterile glass slip (half of a microscopic glass slide; 38 mm × 26 mm), and incubated at 18 °C for 48 h to allow the bacteria to attach to the dish surface. For each strain, 12 replicates were used. Petri dishes were then emptied and rinsed three times gently with 60 ml of AFSW to remove unattached cells. Bacteria that remained firmly attached on surfaces of glass slips were viewed as irreversible attached bacterial biofilms. Next, twenty pediveliger larvae were transferred into individual glass Petri dishes (Ø64 mm × 19 mm height) containing 20 mL AFSW and a monospecific bacterial biofilm. The inducing activity was evaluated by the percentage of metamorphosed individuals (post-larvae) after 48 h. Post-larvae were verified at ×100 magnification under an Olympus stereoscopic microscope. A negative control was included with a clean glass slip instead of the one with attached biofilm. Assays were conducted at 18 °C in darkness with six replicates for each condition.

### Whole-genome re-sequencing

The genomes of wrinkled and translucent variants were sequenced using the whole-genome shotgun method by BGI Co., Ltd. (Shenzhen, Guangdong Province, China) using the Illumina HiSeq 2000 sequencing platform. Genomic DNA was extracted and randomly fragmented using a Bioruptor. The overhangs resulting from fragmentation were converted into blunt ends using T4 DNA polymerase, the Klenow fragment, and T4 polynucleotide kinase. After adding an ‘A’ base to the 3′ ends of the blunt phosphorylated DNA fragments, adapters were ligated to the ends of the DNA fragments. Fragments equal to or smaller than 800 bp were purified by gel electrophoresis, selectively enriched and amplified by PCR. The index tag was introduced into the adapter at the PCR stage, and a library quality test was performed. Finally, the qualified BS library was sequenced. The raw sequencing data were processed after filtering, and the average depth and coverage ratio were calculated. Filtered short reads were assembled using SOAP de novo (version 1.05), and SNPs and InDels were detected based on the aligned result of the assembly sequence and the wild-type reference.

### Construction of in-frame deletion mutants and expression plasmids

In-frame deletion mutants were constructed using our recently developed conjugation-based gene deletion method (Wang et al. 2015). Briefly, the upstream and downstream regions of the target gene open reading frame, which contained the restriction enzyme site at its 5′ ends, were PCR amplified. Recombination plasmids were then constructed by ligation of three DNA fragments. Integration of the recombinant plasmids into the *P. lipolytica* chromosome was carried out by conjugal transfer from *E.coli* WM3064 harboring the suicide plasmid to the strain *P. lipolytica*. The mating agar contained 0.5 % tryptone, 0.1 % yeast extract, half sea water and half distilled water, and 0.3 mM DAP. After allowing 2~5 days for mating, the bacteria were streaked on 2216E medium containing 25 μg/mL erythromycin; positive colonies were visible after 2~5 days. The colonies were then verified by PCR using the Ery-F and Ery-R primers. The deletion mutants were screened by plating the single-crossover strain on 2216E medium containing 15 % sucrose. Further confirmation of the deletion mutant was carried out by PCR using four primer sets SF/SR, SF/LR, SR/LF, and LF/LR. The broad-host-range vector pBBR1MCS was used to express the target genes in *P. lipolytica*. Two genes, *AT00*_*08765* and *AT00*_*17125*, were PCR amplified and ligated to pBBR1MCs-cm after enzyme restriction. The resulting recombinant plasmids were sequenced to confirm their identity using pBBR1MCS-f and pBBR1MCS-r. The recombinant plasmids were transferred into *P. lipolytica* by conjugal transfer from WM3064 harboring the expression plasmid to the strain *P. lipolytica*. The conjugation process was conducted in the same manner as that described above with the exception that chloramphenicol was used for screening.

### Swimming motility assay

The wild-type *P. lipolytica*, its wrinkled variant, and the Δ*08765* mutant were grown in 2216E or SW-LB medium at 25 °C for overnight. One microliter of the overnight cultures were inoculated onto 2216E medium containing 0.3 % agar (Becton Dickinson, USA) and placed at 25 °C for 16 h. Assays were performed with two independent cultures of each strain.

### Congo red and calcofluor assay

The Congo red binding assay was performed according to a previously published method with minor modifications (Nielsen et al. 2011). Colonies grown on 2216E plates were streaked onto 2216E plates containing 8 μg/mL Congo red and incubated at 25 °C for 3 days. The appearance of red or pink colonies on the Congo red plates indicated that Congo red had bound the extracellular matrix material; an increasing depth of color indicated high production levels of the cellulose/curli matrix. For calcofluor assay, cells were collected from SW-LB agar plates and diluted in 2216E broth to an OD_600_ at 4.0 for wild-type and Δ*08765* strains. Calcofluor (15 μg/mL) was added to each sample and was mixed vigorously at 30 °C for 2 h. After 2 h, the mixture was centrifuged at 13,000 rpm for 15 min. Supernatant was collected and was measured at OD_350_. A calcofluor calibration curve was used to determine the cellulose concentration (Fig. S2). Assays were performed with two independent cultures of each strain.

### Transmission electron microscopy

Wild-type *P. lipolytica*, its translucent variant, and the Δ*17125* mutant were grown in 2216E or SW-LB medium at 25 °C. The resulting cultures were collected and diluted in sterile Milli-Q filtered water to an OD_600_ between 0.5 and 1.0. The bacterial suspension was transferred to a formvar-coated copper mesh membrane for 2 min and then covered with 30 g/l phosphotungstic acid at pH 7.0 for another 2 min. After air drying the membrane, the cells were observed and photographed using a Hitachi H-7650 microscope.

### Colanic acid essay

Colanic acid was quantified by measuring fucose according to a previously published method (Zhang et al. 2008). Cells were collected from SW-LB agar plates and diluted in sterile Milli-Q-filtered water to an OD_600_ at 4.0 for the wild-type and the Δ*17125* mutant. Each sample (1 mL culture) was boiled for 10 min and was then centrifuged at 13,000 rpm for 5 min. The supernatant (0.5 mL) was collected, 2.25 mL H_2_SO_4_/H_2_O (6:1 *v*/*v*) was added, and the mixture was heated at 100 °C for 20 min. The mixture (1 mL) was measured at OD_396_ and OD_427_. Next, fresh cysteine hydrochloride (3 % *m*/*v*, 35 μL) was added to the mixture (1.75 mL) and measured again at OD_396_ and OD_427_ after incubation at dark for 1 h. Fucose concentration is calculated by differences of OD_396_ before and after adding cysteine hydrochloride. A l-fucose calibration curve was used to determine the fucose concentration (Fig. S3). Assays were performed with two independent cultures of each strain.

## Results

### Diversification of colony morphology induced in *Pseudoalteromonas* biofilms

**Fig. 1:**
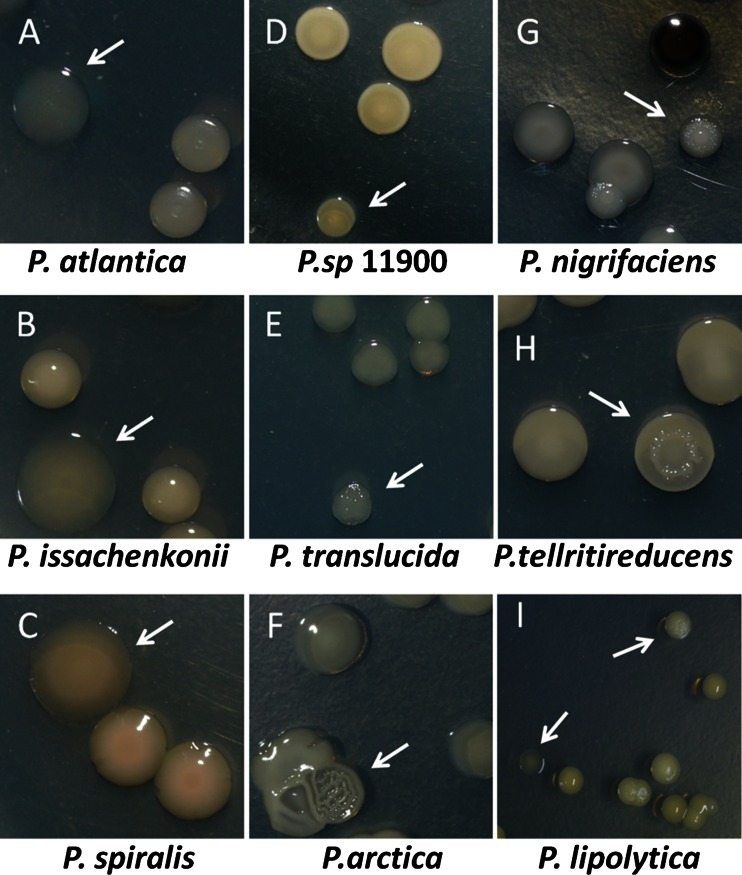
Changes in colony morphology of nine *Pseudoalteromonas* strains during biofilm formation. *Arrows* point to the morphology variants produced by a 14-day-old biofilm at 25 °C in 2216E medium. At least two independent experiments of each strain were tested, and only representative images are shown

To investigate morphological diversification of *Pseudoalteromonas* during biofilm formation, single wild-type cells were propagated in nutrient-rich (SW-LB) medium in static cultures at 25 °C for 7 days and then the biofilm cells were destructively sampled and plated on SW-LB agar plates. We tested twelve strains isolated from diverse habitats (Table S2). Of these, cells from biofilms of *P. atlantica*, *P. issachenkonii*, *P. spiralis*, and *P. sp.* 11900 showed changes in appearance from opaque to translucent (Fig. 1a–d), while cells from biofilms of *P. translucida*, *P. arctica*, *P. nigrifaciens*, and *P. telluritireducens* showed changes in morphology from smooth to wrinkled (Fig. 1e–h). In particular, we observed a relatively high proportion of morphological changes during biofilm formation by *P. lipolytica*; in these cultures, the smooth and opaque wild-type cells generated colonies with wrinkled or translucent morphologies (Fig. 1i). To investigate whether these traits are heritable, at least 20 variants of each type were re-inoculated into fresh medium for three rounds of overnight passaging. None of the variants reverted to the morphology of the wild-type strain, suggesting that the traits were produced by genetic changes. In general, morphological diversification during biofilm formation by *Pseudoalteromonas* was rather common. Therefore, *P. lipolytica* was selected in this study as the organism used to explore genetic basis of the wrinkled and translucent traits during biofilm formation and the impact on larval settlement and metamorphosis.

### Induction of diversified colony morphology in *P. lipolytica* biofilms

**Fig. 2:**
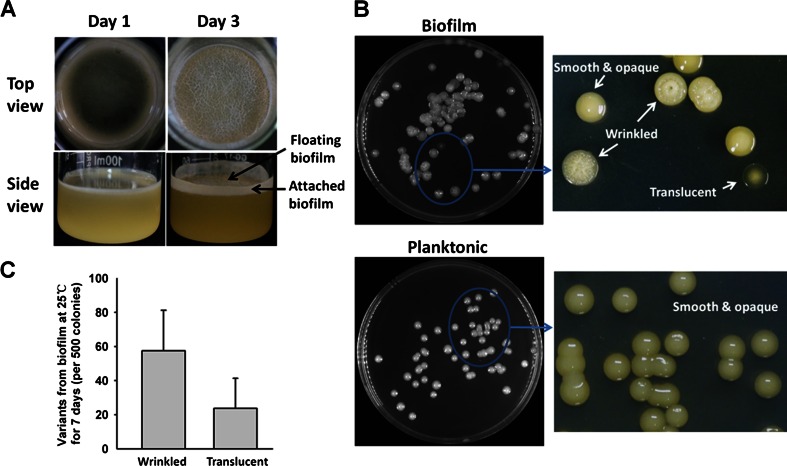
Biofilm formation and colony morphology variants produced in *P. lipolytica* biofilms. **a** Biofilm formed after static culturing in SW-LB medium at days 1 and 3. **b** Colony morphology of day 7 biofilm cells versus day 2 planktonic cells on SW-LB agar plates. **c** Proportion of wrinkled and translucent variants sampled from day 7 biofilm. The data shown are the means of two independent cultures; the *error bars* indicate the standard deviations between independent cultures

When *P. lipolytica* cells were incubated in SW-LB without shaking, they tended to form floating biofilms at the air-liquid interface (also known as “pellicle”) and attached biofilms on solid surfaces (Fig. 2a). After 1 day, a thin pellicle that lacked a complex structure began to form. A stable pellicle with a visible wrinkled surface and attached biofilm on the solid surface were formed after 3 days of incubation (Fig. 2a). After 7 days, extensive morphological diversification was observed when biofilm cells were destructively sampled and plated on agar plates. Most of the phenotypic variants could be assigned to one of the two principal types, wrinkled or translucent (Fig. 2b, upper panel). Of 500 randomly selected cells, 12 ± 5 % appeared wrinkled, whereas 5 ± 3 % appeared translucent (Fig. 2c). Similarly, about 10 % of the cells appeared wrinkled and about 2 % appeared translucent after 7 days of static culturing in 2216E medium (data not shown). In contrast, when the bacteria were cultured in SW-LB or 2216E medium with constant shaking, all planktonic cells appeared smooth and opaque like the wild-type cells (Fig. 2b, lower panel). Therefore, morphological diversification of *P. lipolytica* colonies occurred during the development of biofilms but not during planktonic growth.

### Variants reduce larval settlement and metamorphosis

**Fig. 3:**
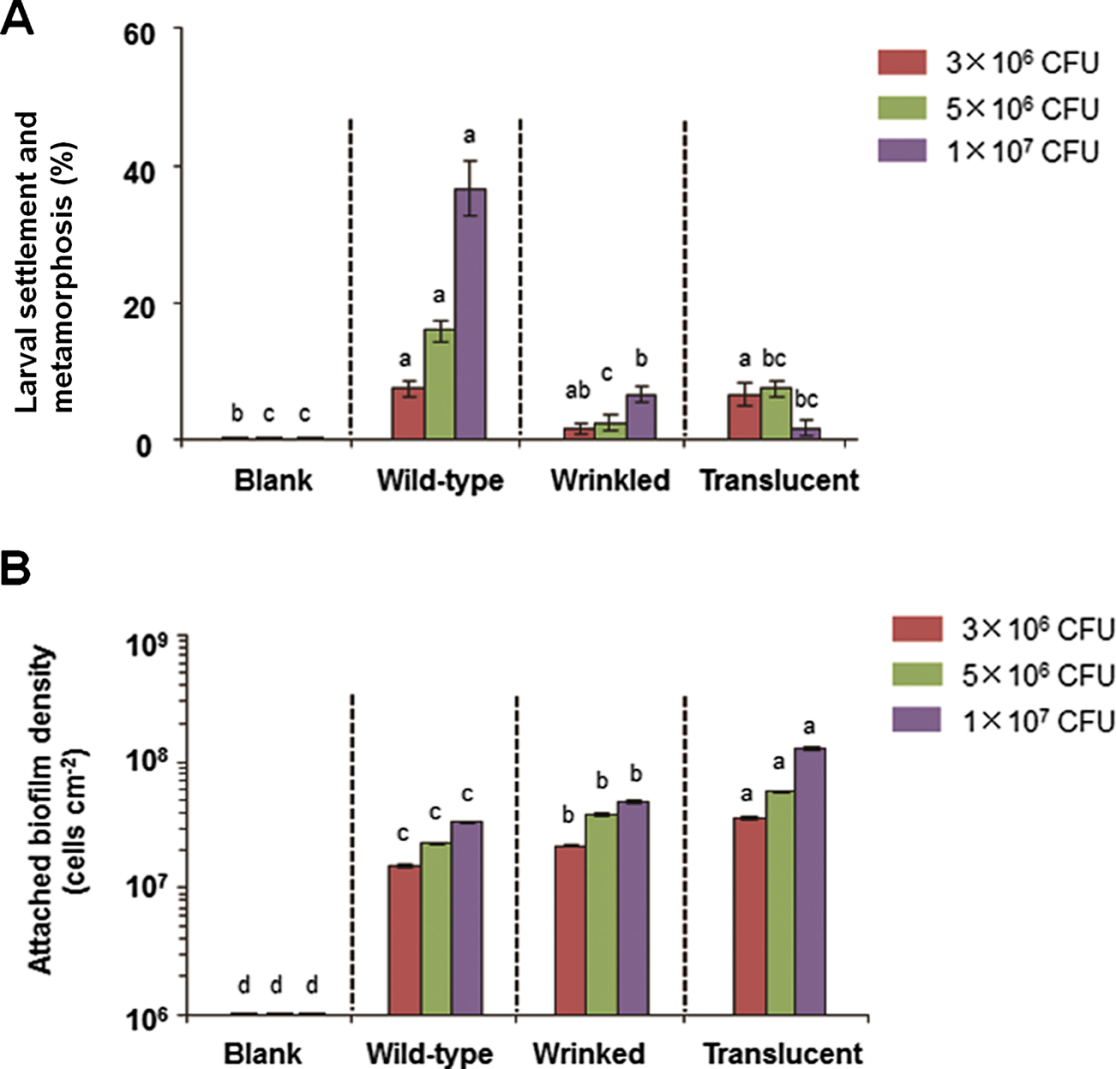
Variants reduce larval settlement and metamorphosis of *M*. *coruscus*. **a** The inducing activities of biofilms of two variant strains and the wild-type strain on larval settlement and metamorphosis with different initial CFU, respectively. **b** Cell densities of the attached biofilms formed by the two variant strains and the wild-type strain on glass slips with different initial CFU, respectively. *Letters in A and B* indicate a significance level of *p* < 0.05 in the same initial CFU

Next, we tested whether biofilms formed by *P. lipolytica* variants affect larval settlement and metamorphosis of the mussel *M. coruscus*. Biofilms of the wild-type strain and the two variants were prepared using the same concentration, and attached biofilms formed on glass slips after a 2-day incubation were used for larval settlement assay (Yang et al. 2013). Results showed that the inducing activities of the attached biofilms formed by the two variants on larval settlement and metamorphosis were significantly reduced when compared to that of the wild-type strain at an initial concentration of 5 × 10^6^ or 1 × 10^7^ CFU/mL (Fig. 3, Kruskal-Wallis test, *p* < 0.001). Larval inducing activities of the attached biofilm formed by the wild-type strain increased with the increase of initial CFU, while a different trend was observed for the translucent variant as there was no significant inducing activity at the highest initial concentration of 1 × 10^7^ CFU/mL (Fig. 3). In addition, we also quantified the cell density of the attached biofilm of each of the three strains, and results showed that the density of attached cells increased with the increase of initial CFU in all three strains (Fig. 3). Hence, the decrease of inducing activities of the two variants was not due to a decrease of cell density in the attached biofilms.

### Whole-genome re-sequencing of the representative variants

Because the observed morphological variation is heritable and because the genetic changes induced during biofilm formation often cluster into one or a few biofilm-related operons within a given species, we used whole-genome re-sequencing based on the whole-genome sequence of *P. lipolytica* to identify the genomic changes that had occurred in the variants (Zeng et al. 2014). Two representative variants, one wrinkled and one translucent, were re-sequenced using the Illumina HiSeq 2000 platform. Genomic DNA was extracted and fragmented into pieces less than or equal to 800 bp in length for library construction. For the wrinkled and translucent variants, 454 and 452 Mb of data, respectively, were produced. By aligning the sequences to that of the reference wild-type genome, 12 point mutations (designated M1 to M12) were identified in the two variants. Of these 12 mutations, seven were non-synonymous mutations, two were nonsense mutations, two were synonymous mutations in the coding region, and one was located in the intergenic region (Table 2). Three of the non-synonymous or nonsense mutations were identified only in the wrinkled variant, and four of the non-synonymous or nonsense mutations were found only in the translucent variant. No large genomic rearrangements were found, suggesting that prophages or genomic islands were not responsible for these two variants. When the cultures were treated with mitomycin C, we also did not find any active phage in *P. lipolytica*. Thus, point mutations rather than genomic rearrangements seem to be the major cause of the phenotypic changes observed in these variants. We next tested whether the non-synonymous and nonsense mutations identified by sequencing caused the phenotypic changes in these two variants.

**Table 2:** Point mutations revealed by whole-genome re-sequencing of the two variants isolated from *P. lipolytica* biofilms

ID	GenBank ID	Gene name	Gene products	Type of mutation	Wild type	Wrinkled	Translucent	Amino acid position
M1	01635	*gcvP*	Glycine dehydrogenase	Nonsyn	C	**A**	C	711
M2	01945	*phrB*	FAD-binding protein	Nonsyn	G	T	T	245
M3	intergenic		Hypothetical protein		G	T	G	
M4	04030		Hypothetical protein	Nonsense	C	C	**T**	145
M5	07185		Hypothetical protein	Nonsyn	G	**A**	G	33
M6	08765		Methylesterase	Nonsense	T	**A**	T	236
M7	09475	*ubiH*	Hydroxylase	Nonsyn	G	G	**A**	327
M8	12970	*glgP*	Maltodextrin	Nonsyn	C	C	**G**	305
			Phosphorylase					
M9	14430		Lipoprotein	Syn	T	T	C	495
M10	15270	*sgaA*	Glyoxalase	Syn	G	T	T	100
M11	16625		Hypothetical protein	Nonsyn	A	T	T	902
M12	17125		Hypothetical protein	Nonsyn	A	A	**T**	177

The non-synonymous or nonsense mutation that is unique in each variant is shown in bold and underlined

### A point mutation in *AT00*_*08765* causes the wrinkled morphology due to cellulose overproduction

**Fig. 4:**
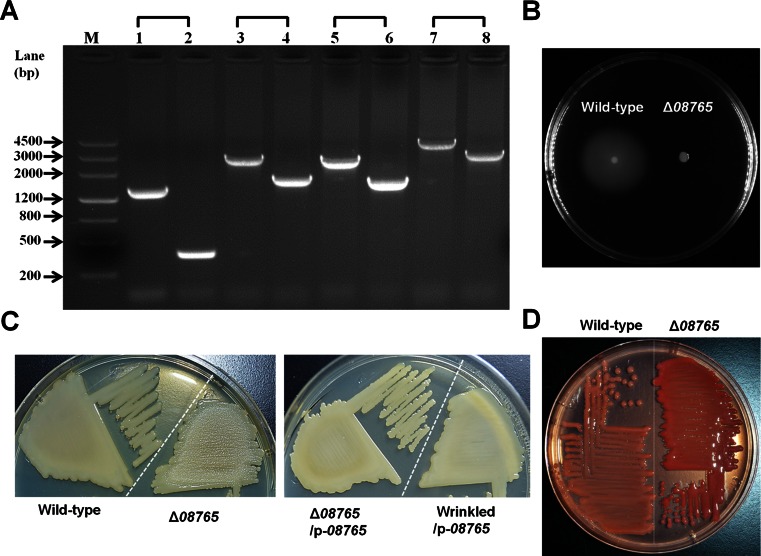
A mutation in *AT00*_*08765* leads to wrinkled morphology. **a** In-frame deletion of *AT00*_*08765* was confirmed by PCR using four sets of primers. *Lanes 1*, *3*, *5*, and *7* used DNA from the wild-type strain, and *lanes 2*, *4*, *6*, and 8 used DNA from the deletion mutant. *Lanes 1* and *2* were amplified using the primer pair 08765-SF/−SR, *lanes 3* and *4* using 08765-SF/−LR, *lanes 5* and *6* using 08765-LF/−SR, and *lanes 7* and *8* using 08765-LF/−LR (Table S1). The expected product sizes were 1491, 2993, 2871, and 4373 bp for the wild-type and 382, 1884, 1762, and 3264 bp for Δ*08765*. **b** Swimming motility test of the Δ*08765* strain versus the wild-type strain. **c** Mutant Δ*08765* showed wrinkled morphology, whereas the wild-type strain showed smooth morphology in SW-LB medium. Complementation of a wild-type *AT00_08765* via plasmid pBBR1MCS-*08765* (p-*08765*) restored the colony morphology of Δ*08765* and the phenotype of the wrinkled variant to smooth. **d** Congo red binding assay of the Δ*08765* strain compared to the wild-type strain. At least two independent experiments of each strain were tested, and only representative images are shown

**Fig. 5:**
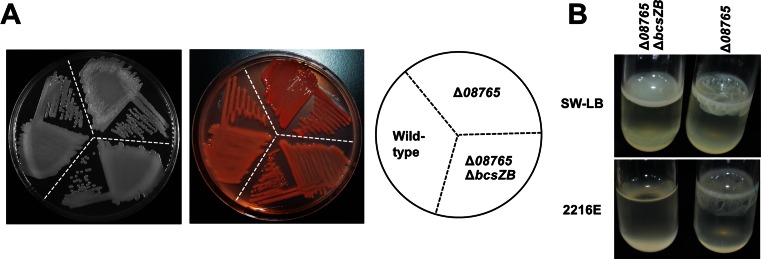
Cellulose production is critical for wrinkled morphology. **a** Mutant Δ*08765* showed a wrinkled morphology, whereas the wild-type and Δ*08765*Δ*bcsZB* strains showed a smooth morphology in SW-LB medium. **b** Floating biofilm formed by the Δ*08765* and Δ*08765*Δ*bcsZB* mutant strains in SW-LB and 2216E medium. At least two independent experiments were performed with each strain, and only representative images are shown in **a** and **b**

Based on whole-genome re-sequencing, three candidate genes, M1, M5, and M6 (Table 2), which were found only in the wrinkled variant, were selected for further verification. We found that M6 (*AT00*_*08765*) encodes a putative methylesterase that shares 30 % similarity (98 % coverage) with the WspF protein of *Pseudomonas aeruginosa.* Thus, an in-frame deletion of the *AT00*_*08765* gene was constructed in *P. lipolytica* to investigate the physiological functions of the corresponding gene product (Fig. 4a); the deletion mutant Δ*08765* completely lost swimming motility (Fig. 4b)*.* When the *AT00*_*08765* gene was deleted, the cells also displayed wrinkled morphology when plated on agar plates (Fig. 4c). Moreover, ectopic expression of *AT00*_*08765* via the plasmid pBBR1MCS*-08765* under the control of its own promoter restored wild-type smooth morphology to both the Δ*08765* mutant and the wrinkled variant isolated from biofilms (Fig. 4c), but the empty plasmid pBBR1MCS failed to do so (results not shown). In addition, the Δ*08765* mutant showed a darker red color on Congo red plates than that of the wild-type strain, suggesting that it produced more cellulose/curli EPS material than was produced by the wild-type strain (Fig. 4d). To test whether the wrinkled morphology is caused by cellulose overproduction, a putative cellulose cluster *bcs* was identified by comparison with the cellulose cluster in *E.coli* (Table S3). When we further deleted two neighboring genes from the *bcs* cluster (*bcsZ-bcsB*) in the *08765* mutant strain, the resulting Δ*08765* Δ*bcsZB* strain no longer showed wrinkled morphology, suggesting that cellulose production is critical in determining the wrinkled morphology of the Δ*08765* mutant (Fig. 5a). In addition, calcofluor assay which is specifically used for cellulose quantification also confirmed that the Δ*08765* mutant bound more calcofluor than the wild-type strain (8.7 ± 1.2 μg/mL versus 4.2 ± 0.8 μg/mL). Moreover, Δ*08765*Δ*bcsZB* formed much less pellicle both in 2216E medium and in SW-LB medium than did the Δ*08765* strain (Fig. 5b). Taken together, these results show that the identified point mutation in *AT00*_*08765* leading to a defective AT00_08765 caused a change from smooth to wrinkled morphology due to the induction of cellulose production.

### A point mutation in *AT00*_*17125* causes translucence due to the reduction of CPS biosynthesis

**Fig. 6:**
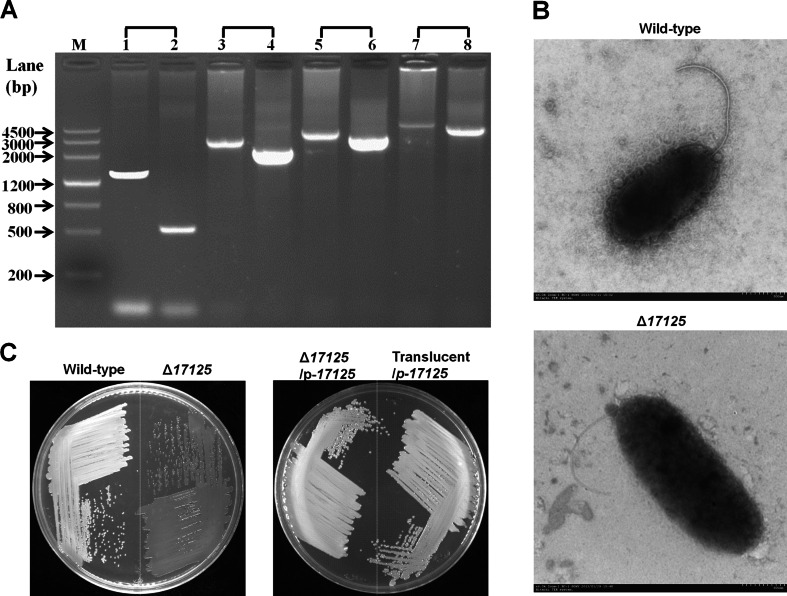
A mutation in *AT00*_*17125* leads to a translucent morphology resulting from a reduction in CPS biosynthesis*.* **a** In-frame deletion of *AT00*_*17125* was confirmed by PCR using four sets of primers. *Lanes 1*, *3*, *5* and *7* used DNA from the wild-type strain, and *lanes 2*, *4*, *6*, and *8* used DNA from the deletion mutant. *Lanes 1* and *2* show DNA amplified using the primer pair 17125-SF/−SR, *lanes 3* and *4* after using 17125-SF/−LR, *lanes 5* and *6* using 17125-LF/−SR, and *lanes 7* and *8* using 17125-LF/−LR (Table S1). The expected product sizes were 1491, 2993, 2871, and 4373 bp for the wild-type and 382, 1884, 1762, and 3264 bp for Δ*08765*. The expected product sizes were 1389, 3030, 3869, and 5510 bp for the wild-type strain and 559, 2200, 2254, and 4680 bp for Δ*17125*, respectively. **b** Morphology of the wild-type and Δ*17125* strains characterized by transmission electron microscopy. **c** Δ*17125* showed a translucent morphology in SW-LB medium. Complementation of wild-type *AT00*_*17125* via plasmid pBBR1MCS-*17125* (p-*17125*) restored the phenotypes of Δ*17125* and of the translucent variant to opaque

Among the four genes (M4, M7, M8 and M12) in which mutations were identified only in the translucent variant, M12 is located within a putative colanic acid capsular polysaccharide biosynthesis cluster ranging from *AT00*_*17080* to *AT00*_*17225* that is homologous to a similar cluster in *Vibrio vulnificus* based on bioinformatics analysis (Table S4). An in-frame deletion of the *AT00*_*17125* gene was successfully constructed in *P. lipolytica* (Fig. 6a). Although the exact function of AT00_17125 remains unknown, transmission electron microscopy revealed that the deletion of *AT00*_*17125* resulted in reduced capsular polysaccharide production (Fig. 6b). Cells of the Δ*17125* mutant displayed translucent morphology when plated on agar plates, and ectopic expression of *AT00*_*17125* via the plasmid pBBR1MCS-*17125* under the control of its own promoter in the Δ*17125* mutant restored the wild-type opaque morphology (Fig. 6c). More importantly, the overexpression of wild-type *AT00*_*17125* in the translucent variant isolated from biofilms also restored the wild-type opaque morphology (Fig. 6c), while the empty plasmid pBBR1MCS failed to do so (not shown). To further test whether *AT00*_*17125* is related to colanic acid synthesis, we measured the colanic acid production between the wild-type and the Δ*17125* mutant by quantifying fucose which is the specific sugar component of colanic acid (Zhang et al. 2008). Results showed that deleting *AT00*_*17125* caused a 12.1 ± 0.5-fold reduction of colanic acid production. Taken together, these results show that the point mutation from A to T, producing a change of the 177th amino acid (Asn to Tyr) of AT00_17125, caused a reduction in the biosynthesis of the colanic acid capsular polysaccharide, leading to a translucent phenotype.

## Discussion

*Pseudoalteromonas* strains have been shown to produce a range of bioactive compounds with antimicrobial, antifouling, and algicidal activities that have attracted scientific and commercial attention (Bowman 2007; Hadfield 2011). In this study, two types of variants with altered colony morphology were isolated during biofilm formation of *P*. *lipolytica*. Using whole-genome sequencing combined with genetic deletion and complementation, we identified the genetic changes in two genes that led to the expression of the wrinkled and translucent phenotypes, respectively. For the wrinkled variant, a point mutation of *AT00*_*08765* causes the wrinkled morphology due to cellulose overproduction. For the translucent variant, a point mutation in *AT00*_*17125* causes translucence due to the reduction of capsular polysaccharide (CPS) biosynthesis. Next, we show that larval settlement of *M. coruscus* was induced by the wild-type strain of *P. lipolytica*, while both variants reduced the inducing activities, suggesting a possible negative correlation between capsular polysaccharide levels and antifouling activity and a positive correlation between cellulose production and antifouling activity. Thus, the study of genetic variants in *Pseudoalteromonas* biofilm can provide insights into a better understanding of antifouling activities of *Pseudoalteromonas* on the molecular level.

Wrinkled or translucent variants are also found during biofilm formation by pathogenic bacteria. The wrinkled variants have been shown to be a result of VPS overproduction in *V. cholerae* (Yildiz and Schoolnik 1999) and a result of Pel and Psl overproduction in *P. aeruginosa* (Hickman and Harwood 2008; Hickman et al. 2005). Here, we showed that cellulose is responsible for the wrinkled morphology variants in *P. lipolytica*. In *P. aeruginosa*, wrinkled variants isolated from cystic fibrosis patients or laboratory biofilm cultures were caused by mutations in the *wspF* or *fleQ* genes (McElroy et al. 2014; Smith et al. 2006). WspF is a methylesterase involved in chemotaxis and is part of the *wsp* (wrinkly spreader) operon, and FleQ is a positive transcriptional regulator of flagellum biosynthesis and EPS production (Porter et al. 2011). Moreover, wrinkled or studded morphology variants can also be produced by inactivation of *wspA* or *wspE* in *Burkholderia cenocepacia*, which is homologous to the *P*. *aeruginosa wsp* operon (Cooper et al. 2014). Because *wspF* variants are commonly found in the biofilms of pathogenic bacteria, we further sequenced the *AT00*_*08765* genes in another 15 wrinkled variants isolated from biofilms in *P. lipolytica*. Of the 15 additional sequenced variants, 12 with similar wrinkled morphology showed mutations in the *AT00*_*08765* gene, including base substitutions and short fragment insertions and deletions (Table S5). All of these mutations caused shifts in the reading frame, translation stops, or amino acid substitutions that might alter or decrease the activity of the AT00_08765 protein. However, no mutation was found in the *AT00*_*08765* gene in three other unidentified mutants with various patterns of wrinkled morphology (Fig. S1). Whether these variants are caused by mutations in *fleQ*, *wspA*, or *wspE* homologs in *P. lipolytica* remain to be explored. In addition, inactivation of *wspF* leads to activation of *wspR* which encodes a GGDEF-domain diguanylate cyclase and, thus, increases intracellular c-di-GMP level (Hickman and Harwood 2008). Here, we found the inactivation of *wspF* in *P. lipolytica* leads to the loss of motility and high production of EPS. These two phenotypic changes can be both caused by a high intracellular level of c-di-GMP (Hickman and Harwood 2008; Hickman et al. 2005). In *P. lipolytica*, we found four genes, *AT00*_*00325*, *AT00*_*20315*, *AT00*_*01115*, and *AT00*_*16440*, containing GGDEF domain, which share >90 % identity (~30 % coverage) with *wspR* of *P. aeruginosa*. Further study is needed to explore whether inactivation of *AT00*_*08765* activates any of the *wspR*-like genes in *P. lipolytica*. Nevertheless, since c-di-GMP can affect cellulose production, manipulating c-di-GMP signaling of marine bacteria via chemical approaches have its potential in control biofouling.

For translucent variants, mutations in *AT00*_*17125* in *P. lipolytica* led to the reduced production of capsular polysaccharide. *AT00*_*17125* is located within a capsular polysaccharide biosynthesis cluster (*AT00*_*17080* to *AT00*_*17190*) that is homologous to that of *Vibrio vulnificus*. In *P. lipolytica*, there are at least 29 genes in this cluster compared to 18 in *V. vulnificus* ( Chatzidaki-Livanis et al. 2006), and *AT00*_*17125* is only present in *P. lipolytica.* The exact function of AT00_17125 is currently unclear. Our TEM results showed that translucent colonies contain markedly less capsular polysaccharide, suggesting that *AT00*_*17125* is either a positive regulator of capsular polysaccharide biosynthesis or a structural gene. To determine whether mutation of the *AT00*_*17125* gene is commonly found in biofilm cells, we sequenced another ten translucent variants isolated from biofilms; however, we did not find any mutations in *AT00*_*17125* in those variants*.* In *V. vulnificus*, inactivation of *wza*, *wzb*, or *wzc* in the CPS cluster leads to a change in morphology from opaque to translucent, enhances biofilm formation, and increases pathogenesis ( Chatzidaki-Livanis et al. 2006; Lee et al. 2013; Nakhamchik et al. 2008; Wright et al. 2001). We also find homologs of these three genes in the genome of *P. lipolytica* (*AT00*_*17180*, *AT00*_*17175*, and *AT00*_*17170*) (Table S4). Indeed, two additional single deletion mutants of *AT00*_*17170* and *AT00*_*17220*, located within the colanic acid capsular polysaccharide, also showed a morphology change from opaque to translucent (data not shown). Thus, we reason that mutations in the rest of the genes within the CPS cluster could also cause translucent morphology.

Taken together, these results suggest that screening of variants using biofilm mode of growth in marine bacteria can be used for the selection of genetic mutants with favorable traits. Recent studies using whole-genome deep sequencing to identify genetic variants in biofilms of *P*. *aeruginosa* (McElroy et al. 2014) and *B*. *cenocepacia* (Traverse et al. 2013) also show that non-synonymous and positively selected mutations govern the within-population bacterial diversification. As many marine bacteria harbor multidrug resistance genes and abundant restriction-modification systems which make genetic manipulation rather difficult (Wang et al. 2015), this selection approach eliminates the need for targeted genetic manipulation or random mutagenesis.

Last but not least, marine bacteria like *Pseudoalteromonas* living in water columns, in sediments, or in association with animal hosts inevitably face with changed environmental factors such as oxygen, nutrients, chemicals, waste products, and signaling molecules (Stocker 2012). Thus, the ocean can play a driving force for the generation of a large repertoire of genetic variants at the population level. Moreover, variants isolated from laboratory-cultured biofilms formed by pathogenic bacteria share similar mutations to those found in clinical isolates during infection (D’Argenio et al. 2007; Drenkard and Ausubel 2002; Woo et al. 2012), suggesting that similar within-population diversification may also occur for marine bacteria living in their marine habitats. To improve understanding of the microbial behavior of various *Pseudoalteromonas* species, including those that survive in extreme marine environments (deep-sea or hydrothermal vents), further studies of the within-population variation of other *Pseudoalteromonas* are warranted.

### Conflict of interests

The authors declare that they have no competing interests.

## Electronic supplementary material

ESM 1 (PDF 502 kb)
