# Supplementary material for: Characterization of self-generated variants in Pseudoalteromonas lipolytica biofilm with increased antifouling activities
Source: Appl Microbiol Biotechnol. 2015 Aug 12;99(23):10127–39. doi: 10.1007/s00253-015-6865-x (PMC4643108; doi:10.1007/s00253-015-6865-x)
Supplement: Supplementary file 1 — (PDF 502 kb) [file 253_2015_6865_MOESM1_ESM.pdf]

SUPPLEMENTARY FILE

Characterization of Self-generated Variants in *Pseudoalteromonas lipolytica* Biofilm with Increased Antifouling Activities

Zhenshun Zeng[1, 2], Xing-pan Guo[3], Baiyuan Li[1, 2], Pengxia Wang[1], Xingsheng Cai[1], Xinpeng Tian[1], Si Zhang[1], Jin-long Yang[3*], Xiaoxue Wang[1*]

[1]Key Laboratory of Tropical Marine Bio-resources and Ecology, Guangdong Key Laboratory of Marine Materia Medica, RNAM Center for Marine Microbiology, South China Sea Institute of Oceanology, Chinese Academy of Sciences, Guangzhou 510301, PR China

[2]University of Chinese Academy of Sciences, Beijing 100049, China

[3]College of Fisheries and Life Science, Shanghai Ocean University, Shanghai, China

*To whom correspondence should be addressed. E-mail: xxwang@scsio.ac.cn; jlyang@shou.edu.cn

Keyword: *Pseudoalteromonas*, biofilm variant, exopolysaccharide, antifouling

Table S1. Sequences of primers used in this study

Primers name	Sequences (5' to 3')
**In-frame deletions**	
17125-up-S	CGCGGATCCACTCATTCTGTGACTTACGG
17125-up-A	ACGCGTCGACACCTCAACAGAGACTACACA
17125-down-S	ACGCGTCGACTCCCTTAGACCCCAATGATA
17125-down-A	ACATGCATGCTAGGCTCTTTGATTGGTTCT
17125-SF	GCTAATGCTATTGAATCGTA
17125-SR	AACATACGCTCGGTTCCTGC
17125-LF	GCTTTTGCTACACTCCTTTC
17125-LR	ATCCTACGGGCTTGAACTGA
08765-up-S	GCTCTAGAAGTAATGAAGGCTCTGATGA
08765-up-A	AACTGCAGTGCCATTAGCACTCTCTTGT
08765-down-S	AACTGCAGCGTGAGACTGGCTGTGGATA
08765-down-A	ACATGCATGCTGCCACCACGACTTTACGAT
08765-SF	ATTGGTCAAGAAGAGGTGGT
08765-SR	ATGACCGCACCTAAAGTTCC
08765-LF	GGTGAAGTAAAAGCCGACAA
08765-LR	TTTCAGCAGGCTCTTCAGGT
bcsZB-up-S	GCTCTAGATCAGAGAGCAGGAAAACAG
bcsZB-up-A	AACTGCAGAGAATAAATGCCCCAAGTAG
bcsZB-down-S	AACTGCAGCATAAAAACCGATGACCAGT
bcsZB-down-A	ACATGCATGCTATCAAGGCTGTTTTGTGTC
bcsZB-SF	GTGGCAGCACTACTTCTTGA
bcsZB-SR	CATATGATTGACCCTCCGAT
17170-up-S	CGCGGATCCGCGACGACAAGCAAAAAGT
17170-up-A	ACGCGTCGACTGCCAATAAACGGGAAAAG
17170-down-S	ACGCGTCGACAATACCGCCTAACCCATCT
17170-down-A	ACATGCATGCCTTCTTGAATACCCCCTGA
17170-SF	GTTATCCACAAGCCAGAGG
17170-SR	AGTGCGTGAACTAAAGGTGC
17220-up-S	GCTCTAGAAGTTTCACAGAAGCCTACG
17220-up-A	AACTGCAGCTTGACAAATTGCCACCAC
17220-down-S	AACTGCAGTGTGAAAATGAGAGCAGCC
17220-down-A	ACATGCATGCCCCGAACATAAGAAGGTAGC
17220-SF	TTACTCGATCTGCCCAACG
17220-SR	ACCCGCAACTGCTGAAGAT
**Complementation**	
17125-pBBR-F	CGGAATTCCGGAAATCAGTTATTAGGTG
17125-pBBR-R	ACGCGTCGACGCATCAACAATGGCAGAGC
08765-pBBR-F	CGGAATTCGATGGTTATTGTGGGTGAG
08765-pBBR-R	ACGCGTCGACTTATCCACAGCCAGTCTCA

Table S2. Information on the twelve *Pseudoalteromonas* strains used for biofilm incubation.

Taxon	Strain	Pigment	Growth temperature	Source
P. atlantica	ATCC 45666	no	5-35 ℃	Seaweed, *Rhodymenia palmata*, Canada
P. issachenkonii	KMM3549	no	4-37 ℃	Thallus of the brown alga *Fucus evanescens*, Kraternaya Bight of the Kurile Islands in the Pacific Ocean
P. spiralis	DSM 16099	no	4-45 ℃	Fluids below a hydrothermal vent flange along the Main Endeavour Segment of the Juan de Fuca Ridge
P. sp 11900	SCSIO_11900	no	4-37 ℃	Layer of coral at a depth of 4 m in the South China Sea
P. translucida	KMM 520	no	4-30 ℃	Seawater collected from the Sea of Japan at a depth of 5 to 8 m
P. arctica	A 37-1-2	no	0-30 ℃	Sea ice, Prydz Bay, Antarctica
P. nigrifaciens	NCIMA 8614	melanin-like	4-30 ℃	Isolate from dairy products
P. telluritireducens	DSM 16098	no	4-45 ℃	Samples of bacterial aggregates taken from Melarie Summit along the Main Endeavour Segment of the Juan de Fuca Ridge
P. lipolytica	SCSIO_04301	no	4-37 ℃	Sediment at a depth of 63 m in the South China Sea
P. sp SM9913	SM9913	no	4-37 ℃	Deep-sea sediment at a depth of 1855 m in the Bohai Gulf, China
P. haloplanktis	TAC125	no	4-35 ℃	Coastal sea water in the vicinity of the French Antarctic station
P. rubra	DSM 6842	red	10-37 ℃	Surface sea water in the Mediterranean Sea near Nice

Table S3. Predicted cellulose polysaccharide biosynthesis cluster of *P. lipolytica* by compared with the *bcs* cluster in *E.coli* K-12 strain W3110 (Serra et al. 2013).

Genbank ID	Gene name	Gene function	Coverage	Identity
AT00_02305	*bcsC*	Cellulose synthase subunit C	82%	30%
AT00_02310	*bcsZ*	Endoglucanase	98%	43%
AT00_02315	*bcsB*	Putative c-di-GMP binding protein	92%	35%
AT00_02320	*bcsA*	Cellulose synthase catalytic subunit containing the PilZ domain	93%	55%
AT00_02325	*bcsQ*	Cellulose synthase containing a putative MinD/ParA-binding domain	18%	39%
AT00_02330	*yhjR*	Hypothetical protein	26%	53%
AT00_02335	*bcsE*	Cellulose synthase subunit E, putative protease	53%	28%
AT00_02345	*bcsG*	Cellulose synthase operon protein	98%	37%

Table S4. Predicted CPS polysaccharide biosynthesis cluster[#] of *P. lipolytica*

GenBank ID	Gene name	Gene function	Coverage	Identity	Homolog
AT00_17080		UTP-glucose-1-phosphate uridylyltransferase			
AT00_17085	*wbfV*	UDP-glucose 6-dehydrogenase	100%	68%	VVMO6_02763
AT00_17090	*wbfY*	nucleotide-diphosphate sugar epimerase	97%	62%	VVMO6_02764
AT00_17100	*wbfU*	lipid carrier:UDP-N-acetylgalactosaminyltransferase	89%	80%	VVMO6_02765
AT00_17105		UDP-glucose 4-epimerase	53%	24%	VVMO6_02775
AT00_17110	*hp4*	colanic acid biosynthesis glycosyltransferase	83%	24%	VVMO6_02772
AT00_17115		heparinase II/III family protein			
AT00_17120		dehydrogenase			
AT00_17125 *		hypothetical protein			
AT00_17130		hypothetical protein			
AT00_17135	*hp3*	hypothetical protein	61%	22%	VVMO6_02773
AT00_17140		hypothetical protein			
AT00_17145		hypothetical protein			
AT00_17150	*wbfT*	UDP-glucose 4-epimerase, glycosyltransferase	95%	61%	VVMO6_02766
AT00_17155		nitroreductase			
AT00_17160	*wecC*	UDP-N-acetyl-D-galactosamine dehydrogenase	90%	30%	VVMO6_02776
AT00_17165	*wbjD*	UDP-N-acetylglucosamine 2-epimerase	92%	29%	VVMO6_02768
AT00_17170 *	*wzc*	tyrosine-protein kinase	97%	47%	VVMO6_02778
AT00_17175	*wzb*	tyrosine phosphatase	100%	54%	VVMO6_02779
AT00_17180	*wza*	polysaccharide export protein	89%	47%	VVMO6_02782
AT00_17185		UDP-phosphate N-acetylglucosaminyl 1-phosphate transferase	100%	68%	VVMO6_02763
AT00_17190	*wbfV*	UDP-glucose 6-dehydrogenase			
AT00_17195		UDP-phosphate N-acetylglucosaminyl 1-phosphate transferase			
AT00_17200		glycosyl transferase family 2			
AT00_17205	*rfaG*	group 1 glycosyltransferase	57%	25%	VVMO6_02771
AT00_17210		teichoic acid biosynthesis protein A			
AT00_17215		hypothetical protein			
AT00_17220 *		colanic acid biosynthesis glycosyltransferase			
AT00_17225		colanic acid biosynthesis protein			

[#] The CPS biosynthesis cluster was identified by comparison with the *cps* cluster in *Vibrio vulnificus*, which lies between VVMO6_02763 and VVMO6_02782 (Chatzidaki-Livanis et al. 2006).

* The deletion of either three genes would lead to translucent morphology in *P. lipolytica*.

Table S5. Mutation of *AT00_08765* in an additional twelve wrinkled variants isolated from *P. lipolytica* biofilms.

Nucleotide position	Type of mutation	Nucleotide change	Consequence
458	Base substitution	C to T	nonsense; translation stop
564	1-bp deletion	C deletion	shift in the reading frame
707	Base substitution	T to A	nonsense; translation stop
875	Base substitution	C to T	missense; proline change to leucine
959-966	8-bp deletion	ATGGTCGT deletion	shift in the reading frame
974	Base substitution	C to A	missense; Alanine change to aspartic acid
1004	4-bp insertion	TTGG insertion prior to T	shift in the reading frame
1061-1105	45-bp deletion	ATGCGGGTTTAGCCAGTGAAAA CCTAGCTCTTAATGATGTTGCTA deletion	shift in the reading frame

Fig. S1. Three unidentified variants with distinct patterns of wrinkled morphology.

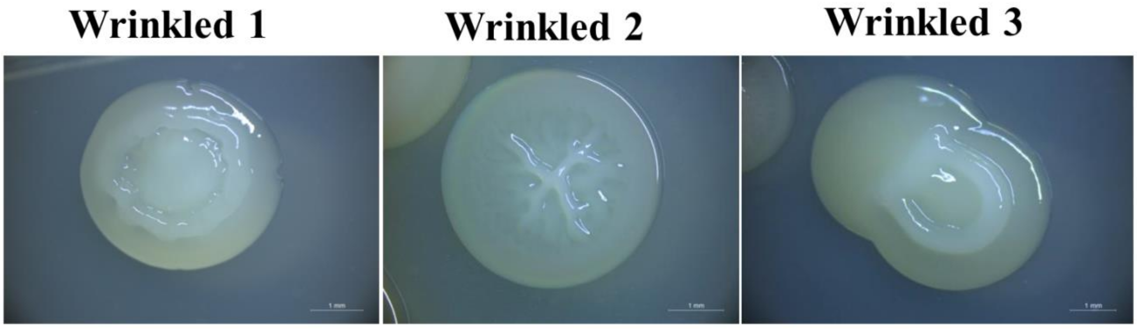

Fig. S2. Standard curve for calcofluor and OD_{350} nm.

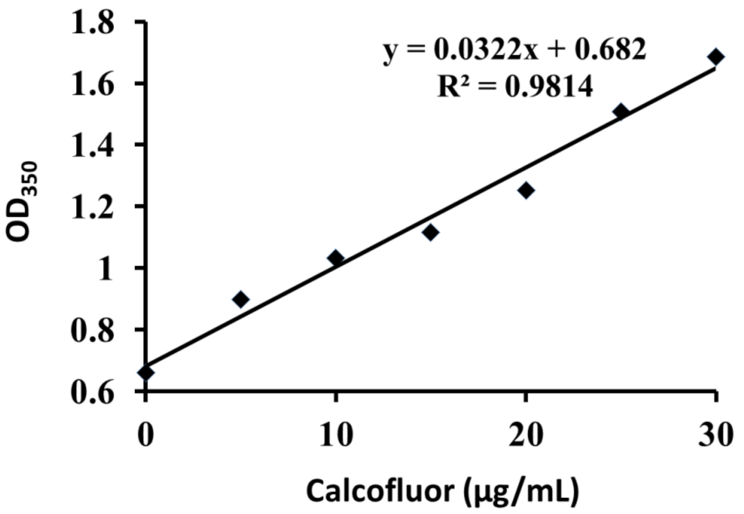

Fig. S3. Standard curve for fucose concentration, calculated as $(OD_{396\text{-}CY}-OD_{396\text{-}CO})-(OD_{427\text{-}CY}-OD_{427\text{-}CO})$

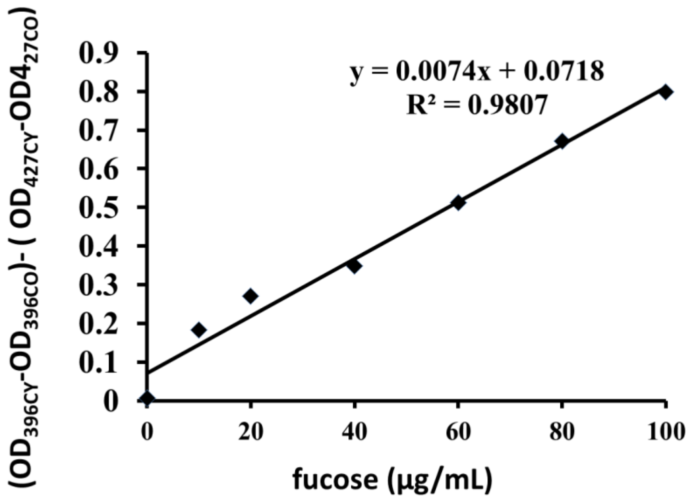

REFERENCE

Chatzidaki-Livanis M, Jones MK, Wright AC (2006) Genetic variation in the *Vibrio vulnificus* group 1 capsular polysaccharide operon. J Bacteriol 188(5):1987-1998

Serra DO, Richter AM, Hengge R (2013) Cellulose as an architectural element in spatially structured *Escherichia coli* biofilms. J Bacteriol 195(24):5540-54
